# Protocol for a family-centered behavioral intervention to reduce early childhood caries: the MySmileBuddy program efficacy trial

**DOI:** 10.1186/s12903-021-01582-4

**Published:** 2021-05-07

**Authors:** Christie L. Lumsden, Burton L. Edelstein, Charles E. Basch, Randi L. Wolf, Pamela A. Koch, Ian McKeague, Cheng-Shiun Leu, Howard Andrews

**Affiliations:** 1 Columbia University College of Dental Medicine, Section of Oral, Diagnostic, and Rehabilitation Sciences, 622 West 168th Street, PH7-322, New York, NY 10032 USA; 2 Dental Medicine and Health Policy & Management at Columbia University Irving Medical Center, Columbia University College of Dental Medicine, 622 West 168th Street, PH7-322, New York, NY 10032 USA; 3 Department of Health and Behavior Studies, Teachers College Columbia University, 525 West 120th Street, Box 137, New York, NY 10027 USA; 4 Mailman School of Public Health, Department of Biostatistics, Columbia University Irving Medical Center, 722 West 168th Street, 6th Fl, Rm 639, New York, NY 10032 USA; 5 Mailman School of Public Health, Columbia University Irving Medical Center, 1051 Riverside Drive, Unit 47, New York, NY 10032 USA

**Keywords:** Dental Caries/diet therapy, Dental Caries/prevention and control, Toothpastes/therapeutic use, Community health workers, Randomized controlled trial

## Abstract

**Background:**

Although largely preventable through diet management and topical fluoride use, early childhood caries (ECC) often progresses to severity that necessitates surgical repair. Yet repair often fails to mitigate caries progression. Needed is an effective behavioral intervention to address underlying behavioral causes.

**Methods:**

This randomized controlled trial will evaluate the efficacy of a behaviorally focused, family-centered intervention, the MySmileBuddy Program (MSB Program), to reduce ECC progression in high-risk preschoolers in New York City. Recruitment will target 858 children ages 24–71 months with ECC and their parents from primary care medical and dental clinics. The study aims to assess the MSB Program’s efficacy to: (1) decrease ECC progression measured 12-months post-randomization; and (2) enhance adoption of a low cariogenic diet and twice-daily fluoridated toothpaste use compared to control group. Potential causal pathways (mediators and moderators) will be explored. The MSB Program equips community health workers (CHWs) with an app that facilitates multilevel risk assessment and provides motivational interviewing-based counseling to inform parents about the caries process, develop personalized goals, and create family-level action plans to achieve targeted behaviors. Social support from CHWs (4 interactions during the 6-month intervention, supplemented by up to 4 in-person/remote contacts throughout the 12-month study period, based on need) is bolstered by automated text messages. Participants will be randomized to a Control Group (paper-based educational handout plus toothbrushes and fluoridated toothpaste for the child) or Intervention Group (MSB Program, two tooth-brushing observations with feedback and instruction, and toothbrushes and toothpaste for the entire family). All children will receive visual ICDAS dental examinations and parents will complete study measures at baseline and 12-months. An incentive up to $150 plus round-trip transit cards ($5.50 value) will be provided.

**Discussion:**

This study hypothesizes that the MSB Program can reduce ECC progression in a high-risk population. Sufficient incentives and a focus on establishing rapport between participants and CHWs are anticipated to mitigate recruitment and retention challenges. If successful, this study will advance the long-term goal of reducing pediatric oral health disparities by demonstrating the efficacy of an acceptable and feasible intervention that shifts attention from dental repair to behavioral risk mitigation.

*Trial registration*: Trial registration was completed on 4/13/2021 through the U.S. National Library of Medicine ClinicalTrials.gov website (Identifier: NCT04845594).

## Background

Dental caries in children under age six years is a persistent, prevalent, and consequential public health problem. More than one-in-five U.S. children ages 2–5 are affected (21.4%) and the disease is disproportionately concentrated in minority and socially disadvantaged populations [1]. Early childhood caries (ECC) has deleterious impacts on the child and family as well as elevating risk for future caries experience in both the primary and permanent dentitions [2–4]. Current treatment regimens focus on surgical repair and pharmacologic suppression [5] while also recognizing the need for preventive guidance to mitigate caries activity [6, 7].

Needed are behavioral strategies that mitigate caries risk for individual children and reduce ECC disparities among child populations. Twice-daily fluoridated toothpaste use [8–10], consumption of a low cariogenic diet, and health-promoting feeding and eating behaviors have both preventive and therapeutic values for young children’s oral health [8, 9, 11–14].

To date, the few US reports of behavioral interventions to reduce ECC have been predicated on theoretical constructs that include Social Cognitive Theory, Health Belief Model, Theory of Planned Behavior, and Self-determination Theory. [12–22]. Three of these studies included both clinical and behavioral outcomes [13, 15, 21] but none analyzed mediators or moderators to describe how or why the intervention was expected to improve ECC-related behaviors or reduce disease progression. What remains unknown are how to increase the adoption and maintenance of salutary behaviors by high-risk families or populations and the reasons why interventions work or fail to work to secure behavioral changes and reduce ECC incidence.

Approaches built on principles of motivational interviewing and chronic disease management have been suggested to promote such positive oral healthful behaviors [23, 24] with mixed results but some notable successes [25, 26].

**Fig. 1:**
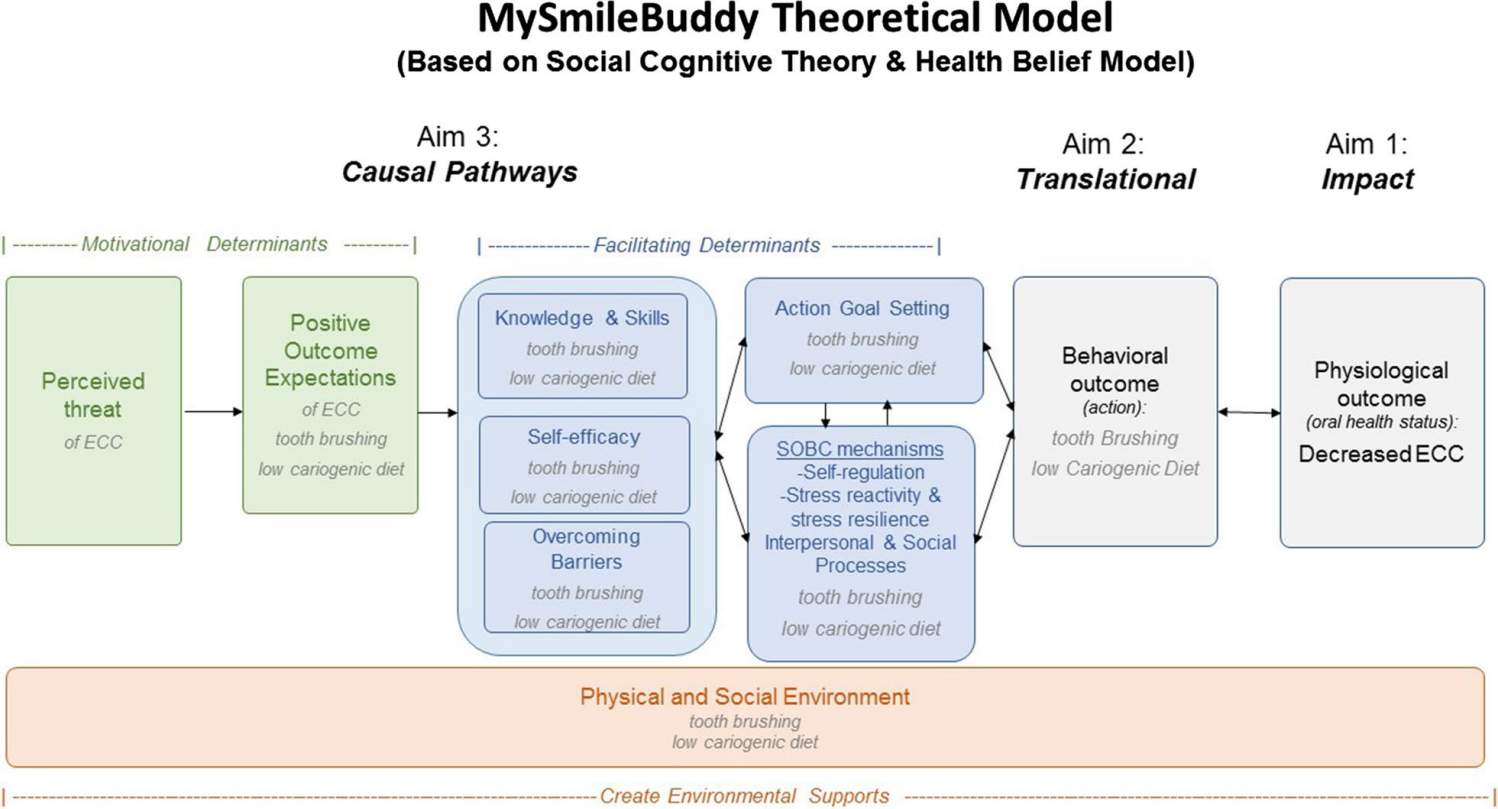
MySmileBuddy program theoretical model. This figure presents the theoretical model that is used to guide the MySmileBuddy Program intervention and analysis of causal pathways through which the intervention exerts (or fails to exert) an influence on targeted behaviors. Mediators included are drawn from MySmileBuddy’s foundation in Social Cognitive Theory and the Health Belief Model. Social Cognitive Theory is based on a wide range of motivating and facilitating determinants. This theory provides extensive guidance on translating motivation into action through its emphasis on action self-efficacy and facilitating determinants. It emphasizes that individuals and their environments mutually influence each other, and so the environment must also be addressed. Health Belief Model focuses on the motivational determinants and the benefits and barriers to taking action and self-efficacy. It is useful for audiences and settings where the emphasis is on health. The included motivational determinants bolster parents’ motivation through increasing perceived threat of caries (both perceived susceptibility and seriousness) and promoting positive outcome expectations. Facilitating determinants are influenced by CHW efforts to increase parents’ ability to act on their motivation by demonstrating skills, enhancing self-efficacy to perform these skills, providing access to tooth brushes and toothpaste, and action goal setting. In addition to these psychosocial theories, three broad evidence-based targets from the Science of Behavior Change (SOBC) program relevant to understanding mechanisms to predict whether participants are successful at action goal setting, are included

One such therapeutic caries counseling behavioral approach to ECC prevention and suppression is the theory-based MySmileBuddy (MSB) Program developed by a multidisciplinary team at Columbia University [27]. An overview of the theoretical model underlying the MSB Program is presented in Fig. 1. Using the MySmileBuddy App to ensure scientific integrity, the MSB Program utilizes Community Health Workers (CHWs) to engage families of young children in home and community sites to educate parents, assess ECC risk factors, establish a personalized behavioral goal, develop an action plan to achieve that goal, and provide ongoing support and follow up. As public health workers who are considered trusted members of the communities they serve, CHWs provide an essential link to health and social services (e.g., housing and legal aid, food assistance, counseling and educational programs, etc.) and support enhanced individual and community capacity by increasing health-related knowledge and self-sufficiency [28]. The MSB Program seeks to both increase motivation and ability to adopt salutary behaviors by providing families with the knowledge, tools, social support, and material supplies to make positive changes, thereby addressing current gaps in knowledge about behavioral management of ECC.

The MSB Program and App were developed with support from the National Institutes of Health (NIH; UL1TR001873, RC1MD004257), evaluated for validity of its risk components [29, 30], assessed for feasibility and acceptability when delivered to a low-income primarily Hispanic population [30–32], and tested through a pragmatic effectiveness trial with families of 1,207 children supported by the federal Center for Medicare and Medicaid Innovation (C1CMS331347). Needed to assess the Program is a randomized controlled trial (RCT) that tests the efficacy of the MSB Program in a high-risk minority population and advances understanding of its mechanisms of action. This report describes the protocol for such an RCT as developed and initiated with support from the NIH National Institute of Dental and Craniofacial Research (R34DE023158, U01DE026739). This RCT was halted in March 2020 by the COVID-19 pandemic just as recruitment began. Since that time, a grant from the Cabrini Foundation of New York City has been secured to refashion the MSB Program for virtual telehealth visits. When and if resumed, this RCT will measure factors shown to influence motivation (perceived threat, ECC contextual knowledge, and positive outcome expectations) and ability to act on motivation (knowledge and skills, self-efficacy, action goal setting, and access to toothbrushes and toothpaste) to initiate and maintain positive oral health behaviors.

## Methods/design

### Aims

The overarching aim of this prospective, single-blinded, RCT is to evaluate the efficacy of the MSB Program – a theory-based, behaviorally focused family-centered intervention – to reduce the incidence of ECC progression in a high-risk population of young children with clinically evident caries. Objectives to fulfill this aim are:

### Impact objective (primary)

To assess the MSB Program’s efficacy versus Control to decrease ECC progression, defined as positive change in number of decayed, missing, or filled teeth (∆dmft > 0) measured 12-months post-randomization. We hypothesize that the MSB Program will decrease ECC progression in Intervention Group participants at 12-months post-randomization.

### Translational objective (secondary 1)

To assess the MSB Program’s efficacy versus Control to enhance adoption of twice-daily fluoridated toothpaste use and consumption of a low cariogenic diet. We hypothesize that the MSB Program will increase adoption of twice-daily fluoridated toothpaste use and consumption of a low cariogenic diet.

### Explanatory objective (secondary 2)

To assess causal pathways (i.e., mediators and moderators) through which the MSB Program influences twice-daily fluoridated toothpaste use and consumption of a low cariogenic diet. We hypothesize that the MSB Program will influence motivation, the ability to act on motivation, and support to maintain oral hygiene and dietary behaviors which may be conditioned by demographic, social, and contextual factors.

### Subjects and setting

This RCT will recruit 858 boys and girls aged 24–71 months with visually evident ECC from Columbia University Irving Medical Center (CUIMC) pediatric medical and dental clinics. They will be randomly assigned equally to Intervention and Control Groups along with their parent or primary caregiver (i.e., the person with legal responsibility for housing and safety, hereinafter referred to as parent). If parents present with more than one age-eligible child, the youngest child will be enrolled.

### Eligibility

Eligible parents must be 18 years or older, have a cell phone with texting capabilities, and plan to remain in the New York City metropolitan area for 12 months after enrollment (Fig. 1). Although open to all racial/ethnic groups, the demographic makeup of the targeted catchment area increases the likelihood that the majority will self-identify as Hispanic. Eligible children must be 24–71 months of age, have a minimum of 12 teeth present, have no disqualifying medical condition (that would limit oral dietary intake, at-home oral hygiene practices, or receipt of oral examinations), and have apparent ECC or Severe ECC (S-ECC) as defined for research purposes [33] on visual examination with light and dental mirror. Suspected ECC/S-ECC at recruitment will be later confirmed upon dental examination by a pediatric dentist trained in the International Caries Detection and Assessment System (ICDAS) if the child demonstrates the presence of one or more decayed (*d*) cavitated or non-cavitated lesions with ICDAS Category 2 or greater, missing (*m*) due to caries on history, or filled (*f)* tooth surfaces in any primary tooth or meets criteria for S-ECC. The ICDAS defines Category 2 as distinct visual change in enamel; Category 3 as localized enamel breakdown due to caries with no visible dentin; Category 4 as underlying dark shadow from dentin; Category 5 as distinct cavity with visible dentin; and Category 6 as extensive distinct cavity with visible dentin [34].

### Recruitment

Parent–child dyads will be recruited in the waiting rooms of one pediatric dental and four pediatric medical clinics of the Columbia University Irving Medical Center (CUIMC) in the Washington Heights neighborhood of New York City. Upon presentation, receptionists will give parents a printed statement substantiating their provider’s endorsement of the study and allowance of waiting room recruitment. Recruitment posters in both English and Spanish will be posted in waiting areas and examination rooms. Recruiters, who are bilingual community health workers (CHWs) will approach parents of young children to explain the study and invite participation. Community Health Workers will obtain informed consent for subsequent eligibility screening, collect parent’s primary and alternative contact information, schedule the screening appointment at the Columbia Community Partnership for Health (CCPH) research and community-engagement facility, and provide the parent with a pre-loaded MetroCard (value $5.50) to cover the cost, if any, of transportation to and from the CCPH. Parents will receive an appointment confirmation call or text message the day before the scheduled CCPH eligibility-screening visit.

Additionally, snowball recruitment will be employed by asking parents to solicit others with age-appropriate children for examination at the enrollment facility. If parents express interest in involving other parents, they will be given a separate recruitment flyer and business card for their friend’s use in contacting an MSB Program CHW.

### Enrollment

At the CCPH screening visit, all parents will be given a gift card valued at $20.00, provided a written report of screening findings, and offered a written referral for routine follow-up dental care along with a printed list of area dentists who treat young children. Parents of children confirmed to have ECC will be invited to participate in the study, asked to provide written informed consent, and informed that they will be randomized to a Control or Intervention Group. Parents who enroll in the study will complete an interview with a CHW, which includes administration of the pre-intervention survey instrument. Their children will receive a visual oral examination with tooth surface-specific charting of caries experience at this baseline visit (T1). Parents will confirm agreement to attend a follow up visit at 12 months (T2) when they will again participate in a CHW interview and survey and their children will have a second study-specific dental examination and charting. Enrolled parents who meet these requirements will be given an additional $30.00 participation incentive. Randomization will be stratified by age (24-48 and 49-71 months) and recruitment site, with approximately equal numbers in each Group using a biased-coin design facilitated by computerized randomization software. Oral examinations will be performed in a consistent manner by one of two study dental examiners blinded to Group assignment.

### Participant incentives

In addition to the MetroCard (value $5.50) to facilitate transit to the CCPH, $20 gift card for presentation at CCPH, and $30 gift card for enrolling in the study, parents who complete the study by participating in T2 examination, interview, and survey will be given gift cards valued at $100 (i.e., up to $155.50 total value).

### Intervention group

At the CCPH visit—in addition to the child’s dental examination, parent’s interview, and completion of the intake survey—Intervention Group parent–child dyads will participate in an observed video-recorded tooth brushing demonstration, receive toothbrushes and toothpaste for the entire family, be provided study-specific educational materials, and receive their CHW’s business card for contact as desired. Based on observation of tooth brushing behaviors, the CHW will offer instruction that promotes skills, helps parents overcome barriers, and bolsters parental self-efficacy. The family will be provided with sufficient toothbrushes and over-the-counter fluoridated toothpaste for the entire family’s use at T1 and replenished quarterly for 12 months (until T2). Printed educational materials will describe the caries process, promote a low cariogenic diet (based on recommended frequency, duration, content, and timing of cariogenic foods and drink consumption), and detail elements of therapeutic tooth brushing (twice daily with appropriate amount of fluoridated toothpaste, by parents, for at least 1 min).

At T1, Intervention Group parents will also engage with a CHW in the first round of the MSB Program. Using the MSB App, positioned to facilitate parents’ engagement, CHWs will involve parents in a conversation that fulfills the first four components of the MSB Program:

1. Education: on caries pathogenesis using videos and models;
2. Caries risk assessment: completion—in any order that feels appropriate to the conversation—of a caries risk assessment that includes considerations of feeding practices, fluoride use, family caries history, beliefs and attitudes regarding caries, and a modified 24-h dietary recall using a unique widget that derives an algorithm-driven dietary cariogenicity score;
3. Goal setting: parent selection of a specific tailored oral health behavioral goal related to an identified high-risk behavior; and
4. Action planning: development of a written action plan that specifies who in the family’s circle will do what, with whom, where, when and how to achieve the stated goal.

The fifth Program component—Follow up and Facilitation—will be achieved over six months through at least three CHW-parent interactions and automated delivery of standardized study-specific text messages. Of the three interactions, at least one will be an in-home visit (or, if necessary, a virtual in-home visit) that will include a timed tooth brushing observation during which CHWs will provide additional guidance and instruction if necessary. Two additional interactions may be conducted by telephone or reciprocal text messaging. Interactions will focus on review and reinforcement of the family’s goal and the family’s progress toward fulfilling their self-defined action plan. Up to four additional interactions with parents may be provided by CHWs if needed to address pressing parental concerns related to food, housing, income, employment, or immigration insecurities by making referrals to appropriate community, social service, and legal aid programs. All interactions will be recorded to analyze possible impacts on study outcomes. The standardized text messages are comprised of 4 rounds of 12–13 messages each over the course of the 6-month intervention, with specific hygiene recommendations (*Brush twice a day; Brush your child’s teeth; Use fluoride toothpaste; and Brush for at least one minute*) and dietary/feeding recommendations (*Don’t buy snacks and drinks that cause cavities; Make water your go-to drink; Make eating time, time to eat; Sit to eat, and Don’t snack all day*) based on behavioral intervention targets. Tooth brushing video recordings, obtained at T1 and T2, will be assessed for quality and duration using the Tooth Brushing Observation System (TBOS) protocol developed by Collett, et al. [35].

CHWs will utilize additional MSB App features including instruction on how to conduct each Program component; a library of culturally-, linguistically-, and literacy-appropriate educational materials suitable for distribution to parents by handout, mail or text; and administrative functions for recordkeeping (case management, tracking families’ progress, and maintaining a log of activities) and data transmission to the study’s Data Coordinating Center (DCC).

If the first four components of the MSB Program are not completed at T1, they will be completed at the in-home visit to be scheduled within one week of the CCHPH visit. If not conducted within one week, the CHW will note challenges and reasons for delay in the built-in electronic CHW log.

### Control group

At T1, parents in the Control Group will complete the same baseline interview and survey as parents in the Intervention Group but will not engage in the MSB Program. Rather than receiving MSB Program-specific educational materials, they will receive materials on caries prevention from the National Institute of Dental and Craniofacial Research. Rather than receiving oral hygiene supplies for the entire family over the course of the one-year study period, they will receive oral hygiene supplies once at T1 and only for the child. At T1, parents will be reminded of their obligation to return one year later for the T2 visit at which time the child’s dentition will be again examined and a videotaped tooth brushing observation will be conducted, recommendations made, and the video subsequently analyzed using the TBOS. The only interim contact Control Group parents will have with Program CHWs will be scheduling of their T2 visit.

The schedule of enrollment, intervention and assessment activities is outlined in Table 1 below following the SPIRIT (Standard Protocol Items: Recommendations for Interventional Trials) guidance for clinical trial protocols.

**Table 1:**
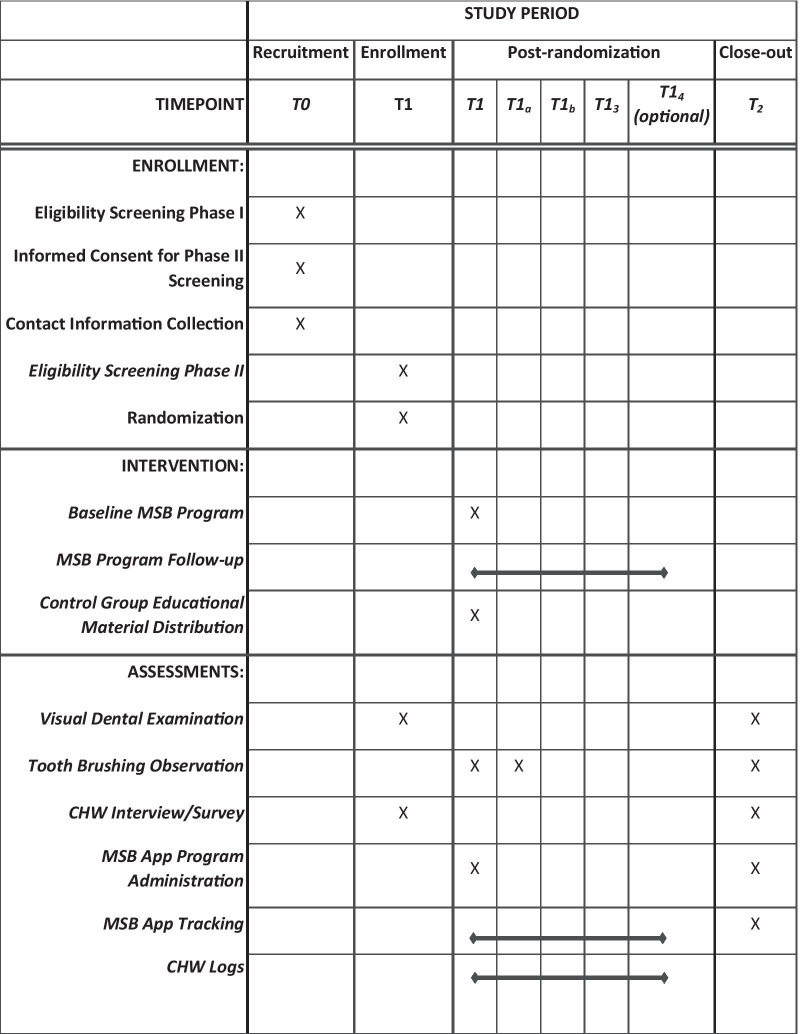
Schedule of enrollment, intervention and assessment activities

### Data management

All data will be securely collected, transmitted and managed electronically in collaboration with the DCC using secure file transfer protocols and a relational database using REDCap Technology®.

**Fig. 2:**
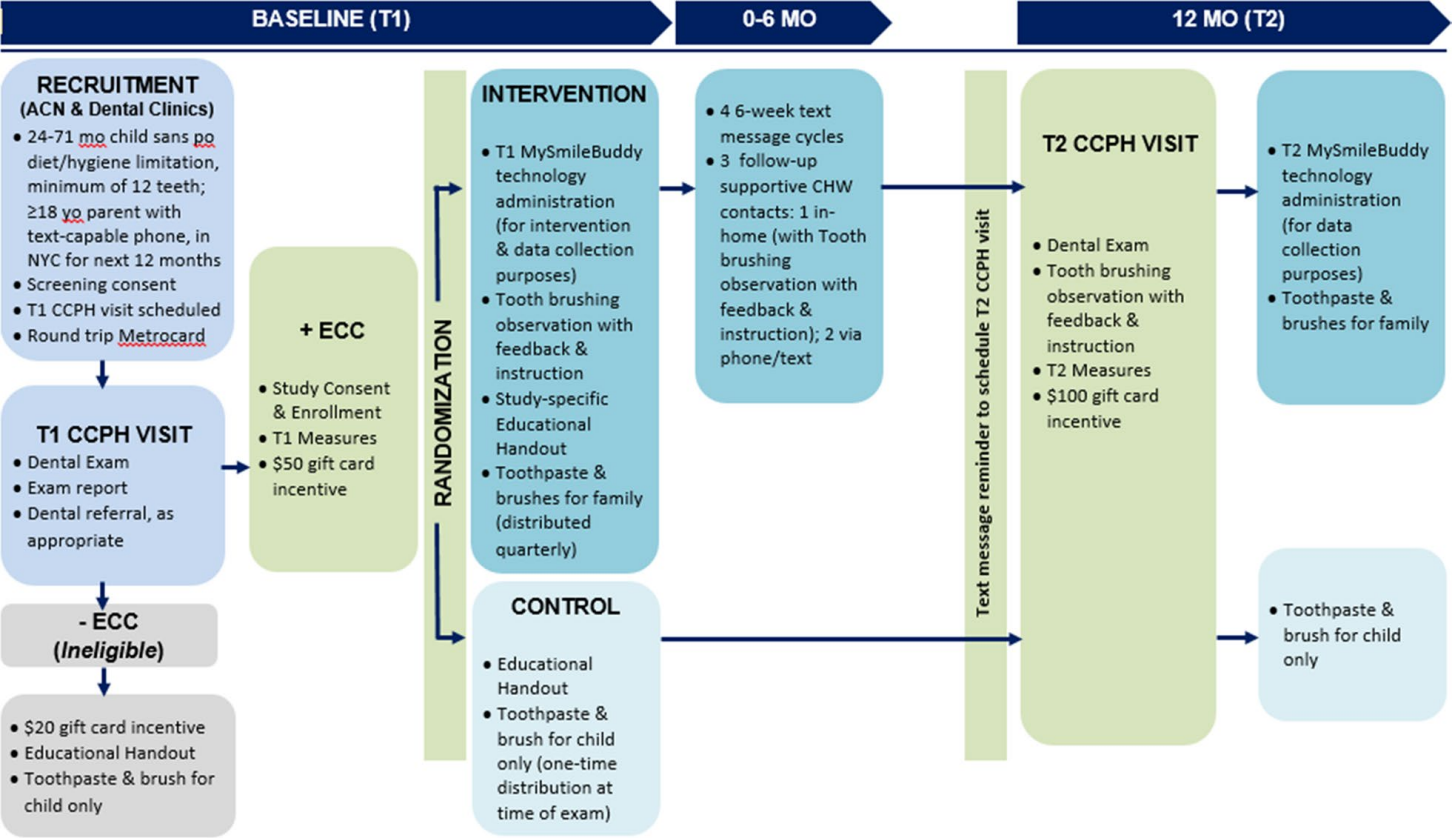
Study schema. This figure presents an overview of study activities and corresponding timeline. The study schema is organized by timepoint: Baseline (T1); 0–6 Months; and 12 Months (T2) post-randomization. The Baseline (T1) timepoint encompasses recruitment, eligibility criteria, enrollment and randomization procedures, and baseline data collection measures. Baseline study activities include both T1 Intervention and Control conditions. The 0–6 Months timeframe represents follow-up activities of the Intervention group condition. Activities represented under the 12 Month (T2) timepoint include final all follow-up data collection measures and activities for both the Intervention and Control group. Intervention and Control Group study activities are described across all timepoints and differences in conditions delineated by arrows depicting flow of study activities

Five primary sources of data will be collected about participants during the 12-month study period:

1. *Clinical dental examination data*: Comparison of surface-level findings using ICDAS classifications at T1 and T2 will allow analyses of caries status, extent, and progression.
2. *Tooth brushing observation data*: TBOS findings for the Intervention Group at T1 and T2 will allow analyses of changes over time while comparison of tooth brushing between Intervention and Control Groups at T2 will allow analysis of difference between toothbrushing efficacy and adherence to recommendations between the Groups.
3. *Interview and Survey data*: Data from CHW interviews and surveys will support evaluation of participants’ sociodemographic characteristics and key motivational (e.g., perceived threat, positive outcome expectations) and facilitating (e.g., knowledge and skills, self-efficacy, action goal setting) determinants that may contribute to explaining mechanisms and putative pathways through which the MSB Program influences the study’s targeted behaviors (Fig. 2).
4. *MySmileBuddy App data*: Data derived directly from the MSB App will describe the Intervention Group’s dietary intake, daily fluoride use, risk profiles, prevention goals, and action plans.
5. *CHW implementation and process data*: Implementation and process data will be collected through electronic forms and logs to track date, time, duration, and disposition of each parent contact and record information on intervention tailoring and referrals for non-dental services.

An overview of data to be collected, data sources, and the time points at which they will be collected is presented in Table 2.

**Table 2:** Data collection overview

Type	Source	Subjects	Timepoint
Clinical dental examination data	Dental exam charting form	All children screened	T1
		Intervention and Control Group	T1, T2
Tooth brushing observation data	Video recording and TBOS Checklist	Intervention Group	T1, T2
		Control Group	T2
	Tooth brushing duration documented by CHW	Intervention Group	Home Visit
Interview/survey data	Baseline Survey	Intervention and Control Group	T1
	12-month Follow-up Survey	Intervention and Control Group	T2
	Experience Survey	Intervention Group	T2
MySmileBuddy technology data	MySmileBuddy Program App	Intervention Group	T1, T2
CHW implementation and process data	Screening Phase I Form	All parent/child dyads approached for recruitment	T0
	Contact Information Form	All parent/child dyads recruited for dental examination eligibility screening	T0
	Screening Phase II Form	All parent/child dyads presenting to CCPH for dental examination eligibility screening	T1
	Visit Log	Intervention Group	T1, home visit, throughout Intervention period, T2
		Control Group	T1, T2
	Outreach Failure Log	Intervention and Control Group	Throughout study period

Various data collection forms will collect additional data including:

1. *Screening phase I form:* used by recruiters to assess eligibility of all parents approached for recruitment and will be used to track study recruitment efforts;
2. *Contact information form:* used by recruiters to collect contact information from parents recruited but not yet screened and enrolled;
3. *Screening phase II form:* used by study staff at the CCPH to report recruited dyads’ eligibility for enrollment, track enrollment, and collect select outcomes measures on dyads who were screened but found ineligible or declined enrollment;
4. *Visit log:* used by CHWs to track implementation of study activities and resources utilized during all interactions throughout the study period;
5. *Outreach failure log:* used by CHWs to track attempts to reach study participants.

Data will also be collected on the MSB Program CHWs at three points in their training: before training to assess baseline CHW measures; post-training to assess training outcomes; and post-intervention to assess CHWs’ study experience.

Figure 2 presents a summary overview of the study schema, outlining study activities at each timepoint.

### Statistical power and data analysis

With power ≥ 80% and 2-tailed alpha = 0.05, a sample size of 300 parent–child dyads per Group is sufficient under various scenarios to detect differences between Control and Intervention Groups (45% vs. 33%, 55% vs. 43%, 65% vs. 53% or 75% vs. 64%) based on a 63.2% one-year caries progression rate among 2–5 year old children treated at the Columbia pediatric dental clinic – a rate consistent with findings from this and other high-risk populations [11, 30, 36, 37]. Accounting for 30% loss to follow up (an estimate higher than experienced in our earlier study), each group will require enrollment of 429 subjects.

### Comparisons

Logistic regression analysis will compare the odds of having ECC progression between the Intervention and Control Group. The analysis incorporates age and recruitment site to reflect randomization on these variables. If there are any significant Group differences in participant characteristics at baseline, a sensitivity analysis adjusting for potential confounder variables in the regression models will be conducted to help interpret the primary analysis finding. To compare the increase in number of dmft/dfs, we will use a repeated measurements analytic approach.

To model the observed count data, we will use a Poisson mixed model (38, Ch.9) that introduces the number of dmft/dfs and considers these counts to be Poisson distributed conditional upon Gaussian random effects. The model includes indicators for time, Group, recruitment site, age, and Group-by-time. The regression coefficient corresponding to the interaction term is the key parameter of interest for assessing the efficacy of the MSB Program as it represents the post- to pre-treatment ratio of the average rate of dmft/dfs between the Intervention and Control Groups. A random effect is included to account for within-subject correlation and for the possibility of heterogeneity among subjects in the ratio of expected dmft/dfs counts before and after randomization. R packages will be used to fit and carry out inference for the proposed models.

Mediation analysis will be used to test of joint significance of the two paths involving a potential mediator, which was found to achieve the best balance of Type I error and statistical power across the 14 reviewed methods [39]. Potential mediators are risk perception for ECC, self-efficacy for tooth-brushing, skills for tooth brushing, self-efficacy for eating a low cariogenic diet, skills for eating a low cariogenic diet, goal setting reflecting self-regulation processes, physical environmental support, social environmental support, knowledge, positive outcome expectations, and ability to overcome barriers to tooth brushing and adoption of a low-cariogenic diet. This allows for expected partial rather than total mediation, a variable will be declared a mediator if and only if both the test of the regression coefficient of the explanatory factor on the mediator *and* the test of the coefficient of the mediator on the outcome variable are both significant at level alpha = 0.05, two-tailed.

Missing outcome data due to subject dropout can introduce bias that results in misleading inferences since subjects may drop out for reasons related to the outcome of interest. We will use random effect logistic regression models to examine whether missing data are completely random, missing at random, or non-ignorable (38, Ch. 13). In the first two cases our proposed approach based on maximum likelihood estimation is still valid, and multiple imputation of missing outcomes is also valid, as long as the models are correctly specified. If the missingness mechanism is found to be non-ignorable we will use tipping point analysis as sensitivity analysis to assess the potential impact of missingness on our results.

## Discussion

Over six years of preliminary research [29, 30], an NIDCR-funded planning grant (R34DE023158), and refinement in collaboration with NIDCR (U01DE026739), protocols were developed to minimize recognized large RCT challenges in participant recruitment, enrollment, and retention. Reaching the study’s targeted enrollment is achievable at a pace of 8–9 dyads enrolled weekly over 24 months given that more than 800 age-eligible children visit the recruitment sites each month, at least half of 4–5 year olds and one-quarter of 2–3 year olds in the target population experience ECC, and a preliminary study of 108 families found a 95.6% acceptance rate without financial incentive [30]. While the preliminary study involved only one recruiter 2.5 days/week for five months, this study will engage three CHW recruiters and three support staff five days each week for 24 months and will provide financial incentives. Recruitment and enrollment rates will be monitored weekly and additional resources invested as necessary.

This study encourages retention through financial incentives, user-friendly technology, rapport between CHWs and parents, assistance to families to address social service needs, and an approach that is non-judgmental and encouraging. Initial and semiannual CHW training will help ensure that study protocols are well understood and that appropriate communications and referral skills are established. Adherence to study protocols will be monitored for fidelity by a CHW supervisor and the Study Team through recurrent retraining and bi-weekly meetings that identify and address problems as they arise and weekly review of CHW logs and DCC data for timeliness, completeness, and accuracy (e.g., reported data that are within allowable limits). Corrective action plans will be developed and implemented as needed.

The primary assessment of intervention effects will use an intention-to-treat approach. Implementation data will allow testing of dose–response effects if some subjects do not complete all intervention activities thereby enabling assessment of potential moderating effects attributable to delivery of varying levels of intervention intensity.

Through these efforts, the study team seeks to maximize its ability to meet the study’s *Impact*, *Translational*, and *Exploratory* objectives and build understanding of oral health behavior change that holds promise to improve individual children’s oral health and the oral health of socially disadvantaged populations.

## Data Availability

Not applicable.

## Items from world health organization trial registration data set

**Table:**

Data category	Information
Primary registry and trial identifying number	Trial registration was completed through the U.S. National Library of Medicine ClinicalTrials.gov website (Identifier: NCT04845594)
Date of registration in primary registry	4/13/2021
Secondary identifying numbers	Not Applicable
Source(s) of monetary or material support	National Institute of Dental & Craniofacial Research of the National Institutes of Health (U01DE026739)
Primary sponsor	National Institute of Dental & Craniofacial Research of the National Institutes of Health (U01DE026739)
Secondary sponsor(s)	Not Applicable
Contact for public queries	*CLL,* PhD, MS, RD, CDN
	Columbia University College of Dental Medicine
Contact for scientific queries	*CLL,* PhD, MS, RD, CDN
	Columbia University College of Dental Medicine
Public title	Family-centered Behavioral Intervention to Reduce Early Childhood Caries: The MySmileBuddy Program efficacy trial
Scientific title	Randomized Efficacy Trial of MySmileBuddy, a Family Centered Behavioral Intervention to Reduce Early Childhood Caries
Countries of recruitment	United States
Health condition(s) or problem(s) studied	Early childhood dental caries
Intervention(s)	Intervention: Tooth brushing observation at T1 and during home visit with feedback and instruction immediately following observations; two administrations of the MSB technology (i.e., iPad app) by CHWs at the CCPH site (~ 30 min, face-to-face contact at T1 and T2, with T2 administered for data collection purposes only); six months of automated text messages (4 rounds of messages with approximately 50 messages each regarding key intervention topics: Brush twice a day; Brush your child’s teeth; Use fluoride toothpaste; Brush for at least one minute; Don’t buy snacks and drinks that cause cavities; Make water your go-to drink; Make eating time, time to eat; Sit to eat, don’t snack all day); one in-person, in-home visit and two telephone/text message follow-ups by CHWs; up to 4 additional supportive CHW contacts, in-person or by phone/text message are allowed, as needed, to address urgent social issues and will be recorded to discern potential impact on study outcomes
	Control: Mimic standard of care, providing paper-based educational handout plus toothbrushes and fluoridated toothpaste for the child at T1
Key inclusion and exclusion criteria	Ages eligible for study: well-children 24–71 months of age with a minimum of 12 teeth and apparent early childhood caries (ECC) or Severe ECC (S-ECC) as defined for research purposes; parents/caregivers ≥ 18 years of age with text-capable phone planning to reside in the New York area for next 12 months
	Exclusion criteria: Children with a disqualifying medical condition (that would limit oral dietary intake, at-home oral hygiene practices, or receipt of oral examinations)
Study type	Single-blind (clinical outcomes assessor) randomized controlled trial with randomization stratified by age (24 – 48 and 49 – 71 months) and recruitment site, with approximately equal numbers in each Group using a biased-coin design facilitated by computerized randomization software
Date of first enrolment	Pending
Target sample size	858 parent/child dyads
Recruitment status	Pending
Primary outcome(s)	Change in number of decayed, missing, or filled teeth (∆dmft > 0) or surfaces (∆dfs > 0)) measured 12-months post-randomization
Key secondary outcomes	Change in twice daily therapeutic toothbrushing (twice daily with appropriate amount of fluoridated toothpaste, by parents, for at least 1 min) and consumption of a low cariogenic diet (based on recommended frequency, duration, content, and timing of cariogenic foods and drink consumption)

## Abbreviations

**CCPH**: Columbia Community Partnership for Health
**CHW**: Community Health Worker
**DCC**: Data Coordinating Center
**dmft/dfs**: Decayed, missing or filled teeth/decayed or filled surfaces
**ECC**: Early childhood caries
**ICDAS**: International Caries Detection and Assessment System
**MSB**: MySmileBuddy
**NIDCR**: National Institutes of Dental and Craniofacial Research
**NIH**: National Institutes of Health
**RCT**: Randomized controlled trial
**S-ECC**: Severe early childhood caries
**TBOS**: Toothbrushing observation system
